# Transient Knockdown of Tyrosine Hydroxylase during Development Has Persistent Effects on Behaviour in Adult Zebrafish (*Danio rerio*)

**DOI:** 10.1371/journal.pone.0042482

**Published:** 2012-08-03

**Authors:** Isabel Formella, Ethan K. Scott, Tom H. J. Burne, Lauren R. Harms, Pei-Yun Liu, Karly M. Turner, Xiaoying Cui, Darryl W. Eyles

**Affiliations:** 1 Queensland Brain Institute, University of Queensland, Brisbane, Queensland, Australia; 2 School of Biomedical Science, University of Queensland, Brisbane, Queensland, Australia; 3 Queensland Centre for Mental Health Research, Brisbane, Queensland, Australia; University of Iowa, United States of America

## Abstract

Abnormal dopamine (DA) signaling is often suggested as causative in schizophrenia. The other prominent hypothesis for this disorder, largely driven by epidemiological data, is that certain adverse events during the early stages of brain development increase an individual's risk of developing schizophrenia later in life. However, the clinical and preclinical literature consistently implicates behavioural, cognitive, and pharmacological abnormalities, implying that DA signaling is abnormal in the adult brain. How can we reconcile these two major hypotheses underlying much of the clinical and basic research into schizophrenia? In this study we have transiently knocked down tyrosine hydroxylase (TH, the rate limiting enzyme in DA synthesis) gene expression in the early stages of brain development in zebrafish using morpholinos. We show that by adulthood, TH and DA levels have returned to normal and basic DA-mediated behaviours, such as locomotion, are also normal. However, when they were exposed to a novel environment the levels of freezing and immediate positioning in deeper zones were significantly reduced in these adult fish. The neurochemistry underlying these behaviours is complex, and the exact mechanisms for these abnormal behaviours remains unknown. This study demonstrates that early transient alterations in DA ontogeny can produce persistent alterations in adult brain function and suggests that the zebrafish may be a promising model animal for future studies directed at clarifying the basic neurodevelopmental mechanisms behind complex psychiatric disease.

## Introduction

Schizophrenia is a chronic psychotic disorder that affects 0.5–1% of the population. Although the onset of schizophrenia is typically in late adolescence or early adulthood [1], [2] there is convergent evidence from various research fields, including epidemiology, neuroimaging and post-mortem analysis that schizophrenia is both a developmental disorder [3], [4], [5] and that patients have abnormalities in dopamine (DA) signaling [6]. According to the neurodevelopmental hypothesis of schizophrenia, disruptions during early neural development lead to altered synaptic transmission and plasticity and have a fundamental impact on the etiology of schizophrenia long before the disorder is clinically expressed [7], [8]. Animal models for this disease have been developed in rodents and virtually all of these models reveal behavioural, cognitive or pharmacological abnormalities reflective of aberrant dopamine (DA) signaling (http://www.schizophreniaforum.org). In particular those animal models that have been developed specifically to address known epidemiological risk factors for schizophrenia, such as prenatal vitamin D deficiency or prenatal infection, reveal very early changes in DA development [9], [10], [11], [12]. As a result, early disruptions to DA systems have recently been proposed as an initial common pathway prior to disease onset [13]. However, while there is ample evidence linking abnormal DA signaling and schizophrenia, little is known about how early adverse events in development could alter DA physiology.

In mammals the dopaminergic system is associated with behaviours as diverse as locomotion, motivation and reward-based learning [14] and this appears to be conserved in zebrafish (*Danio rerio*). Adult fish exhibit a broad spectrum of behaviours, such as bottom-dwelling, freezing and erratic movement that are affected by intrinsic DA status [15], [16], [17]. Several paradigms used to assess behaviours influenced by DA in rodents have also been translated into zebrafish studies, such as the open-field and place preference. Another extensively studied behaviour in adult zebrafish is the novel diving test. All three tasks examine the ethologically normal conflict between preferences for a protected area (e.g., black substrata at the bottom of open waters) and innate motivation to explore novel environments in fish [16], [18], [19], [20], [21], [22], [23], [24], [25], [26].

In the current study we have used morpholino oligonucleotides (MOs) to transiently knock down DA production in the developing larval brain to investigate how such early alterations in DA production could affect long-term brain function in adult zebrafish. Recent studies using rat, mice, zebrafish, nematodes and fruit flies have begun to identify several highly conserved genes as potential players in the development and specification of dopaminergic neurons, including *ptx3*, *lmx1b*, *nr4a2*, *otp* and *th* [27], [28], [29], [30], [31], [32]. As a first step we have used MOs directed against the rate-limiting enzyme in DA synthesis, tyrosine hydroxylase (*th*), which catalyses the hydroxylation from L-tyrosine to L-DOPA, the precursor of DA. Our hypothesis was that transient impairment in the ability of the zebrafish larvae to synthesize DA early in development will lead to persistent alterations in adult brain function and behaviour.

## Materials and Methods

### Zebrafish maintenance and tissue collection

Wild-type zebrafish (Tupfel Longfin strain) embryos and larvae were bred and maintained under standard conditions [33]. Embryos were staged according to Kimmel et al. [34] and reared at 28°C. Adult zebrafish (90–110 days of age) used for behavioural studies were housed in groups of 6 in 3 L tanks with constant water flow. The University of Queensland Animal Ethics Committee approved all procedures, under the guidelines of the National Health and Medical Research Council of Australia. For studies that required brain tissue, larvae and adult fish were euthanized by submersion in ice water (5 parts ice/1 part water) for 15 minutes.

### MO-mediated gene knockdown

MOs (GeneTools, LLC, Philomath, OR, USA) were designed targeting the splice donor sites of exon 2 and exon3 of the zebrafish *th1* gene (ENSDARG00000030621): *th1*-MO1 (targets exon2: 5′- AAAACATTATGTTAGCCTACCTCGA -3′) and *th1*-MO2 (targets exon3: 5′- CAGGTTAACAGACTTACATTTGACC -3′). At a 1–2-cell stage, embryos were injected directly into the yolk with 6 ng of MO as well as a standard control MO (5′- CCTCTTACCTCAGTTACATTTATA -3′). Reductions in *th* mRNA were confirmed by real-time-PCR (oligonucleotide primer pair that did not span the same exon-intron boundaries used in designing the morpholinos, *th1*: 5′ GCTCTAAAAGCCCTGCGCT 3′ and 5′ TTTGGTGACAAGATGATGGCA 3′). The standard zebrafish housekeeping *elf1a* was used as a reference gene. *th1*-MO-1 and *th1*-MO2 exposure had no effect on either larvae or adult fish survival compared with standard control MO.

### TH Immunohistochemistry

6 dpf larvae were fixed in 4% paraformaldehyde overnight at 4°C. Larvae were transferred into phosphate-buffered saline, and their brains were dissected and stored at 4°C. Whole larval brains were processed as described by Westerfield (2000) [33] and incubated overnight with primary mouse monoclonal anti-TH (1/500, Millipore, California, USA), which specifically detects TH1-expressing CA neurons [35], [36], [37], [38]. Th-positive cells were visualized by Alexa555-conjugated secondary goat anti-mouse antibody (Invitrogen). Serial Z-stacks of larval brains were recorded using a Zeiss LSM 510 META confocal microscope.

### TH Western blot

To assess TH protein levels, larvae were collected at 6 dpf, de-yolked in cold Ringer's solution (116 mM NaCl, 2.9 mM KCL, 1.8 mM CaCl2 and 5 mM HEPES, pH 7.2), homogenized and 10 larvae/treatment pooled for analysis. All larvae experiments were performed in triplicate. Individual adult brains were dissected, homogenized in Ringer's solution and centrifuged (n = 12/group). Supernatants were collected for protein quantification (BCA™ protein assay, ThermoScientific, USA) and Western Blot analysis. Twenty micrograms of total protein for each sample was loaded on each lane on a 4–12% Bis-Tris gel (NuPAGE**®**, Invitrogen, USA) and SDS-PAGE was run at constant 150 V for 1 h. Each gel was transferred to PVDF membrane (Millipore, USA) using wet-transferring method (Mini Trans-Blot**®**, Bio-Rad Life Science Research, USA). Transferred membranes were blocked in 10% skim milk with 1× PBST at room temperature for 1 h and incubated with the same mouse monoclonal anti-TH antibody used in immunohistochemical studies (1∶1,000, Millipore) at 4°C overnight. Membranes were then washed with 1× PBST and incubated with Alexa 680 conjugated goat anti-mouse antibody (1∶20,000, Invitrogen) at room temperature for 1 h. Membranes were washed again with 1× PBST for 3 times and then scanned under Odyssey imager (LI-COR Bioscience, USA). Densities of each protein band on scanned images were semi-quantified using ImageJ (National Institute of Health, USA).

### Measuring dopamine (DA) levels

For 6 dpf larvae heads were removed and 10 larvae/treatment pooled for analysis. All larvae experiments were performed in triplicate. Adult brains were dissected and prepared individually (n = 12 per group). Tissue was immersed in 100 µl of 0.1 M perchloric acid containing the internal standard, 50 ng/ml deoxyepinephrine (DE) and kept on ice. Samples were dispersed by sonication then centrifuged and the supernatant filtered (0.22 µm, 4 mm) prior to injection onto a HPLC system (Model 1100, Agilent Technologies, Inc. CA), using a Sunfire C18 5 um 4.6 mm×150 mm column (Waters Corporation, MA); and a Coulochem III electrochemical detector (ESA Laboratories, Inc. MA). The mobile phase consisted of 12% acetonitrile/75 mM potassium dihydrogen phosphate buffer with the addition of 1 mM EDTA and 1.4 mM octane sulfonic acid adjusted to a pH of 4.1. The conditioning cell (Model 5020, ESA Laboratories, Inc. MA) set to +350 mV and the analytical cell (Model 5014B, ESA Laboratories, Inc. MA) was operated at −150 mV at the first electrode and +250 mV at the second. Peak-height ratios for DA, relative to the internal standard (DE), were calibrated against standard curves. Values were then corrected for dilution.

### Behavioural assessment

**Figure 1:**
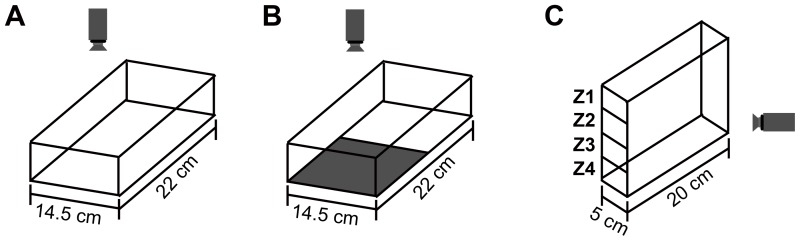
The open field, place preference and the novel diving tank. Illustration of the open-field tank (A), place preference (B), and the novel diving test tank (C) with specific dimensions. Virtual divisions in the open-field tank and in four zones of the diving tank (Z1/top – Z4/bottom) were used to evaluate fish positioning.

Prior to testing adult fish were brought from the fish facility to the behaviour room in their housing tanks and given 1 h to acclimatize. Fish were tested in two rectangular tanks of different size depending on the behaviour assessed: I) open-field tank (22 cm×14.5 cm×5 cm to water surface; camera from above) (Figure 1A), II) place preference tank (22 cm×14.5 cm×5 cm to water surface; the background of the tank was divided into white and black compartments of the same size, camera from above) (Figure 1B), III) diving tank (20 cm×5 cm×12 cm to water surface; camera from aside) (Figure 1C). Fish were filmed during free swimming and quantitative analyses were performed for exploration, thigmotaxis (tendency to remain close the walls of the tank), scototaxis (preference for dark environments), bottom-dwelling, freezing bouts (movement <0.2 cm/s) and intra-session habituation.

All fish tested were placed individually in the centre (open-field tank and place preference tank) or zone 1 of (diving tank) of the novel environment and locomotion and position recorded. To assess intra-session habituation the behaviours displayed during the 1^st^ minute were compared to those displayed during the 10^th^ minute. Behavioural activity was recorded with a high-speed camera (Fastec Imaging, Germany) at 75 fps. To avoid test order effects, tanks were cleaned with 70% ethanol and rinsed after each trial. Locomotor activity and position was assessed automatically using Ethovision XT Version 5.1 (Noldus Information Technology, Wageningen, The Netherlands). To quantify vertical positioning, the diving tank was divided into four equal horizontal zones (zone1 (top), zone2, zone3, zone4 (bottom)). To quantify horizontal position the open tank was divided into two zones, the area along the walls (37.5% of the total area) and the center (62.5% of the total area). Freezing was assessed using automated immobility measures in Ethovision, which have previously been demonstrated to correlate highly with manually-scored freezing behaviour [39]. Percent freezing was defined as the proportion of samples in which the fish moved less than 0.2 cm/s per sample. These tests were selected because they are known to be sensitive to disruptions in DA signaling [15], [16], [40].

### Statistical analysis

All biochemical measurements such as mRNA, protein expression or DA content were examined by one-way ANOVA with post-hoc Dunnett's tests for multiple comparison. All behavioural assessments were analyzed using the SPSS software package (Release 12.0.1, SPSS Inc., Chicago, Illinois). Data were either analysed using one-way ANOVA followed by Bonferroni correction or using repeated measures ANOVA where appropriate. Data are presented as mean ± SEM. A minimum of 17 adult fish per group was tested. There were no significant differences detected between genders, thus all data are presented pooled for sex.

## Results

### MO-mediated loss of *th1* function perturbs dopaminergic neuron development in zebrafish

**Figure 2:**
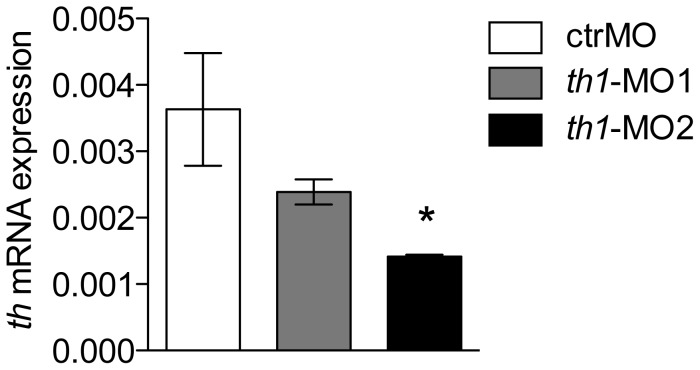
Efficiency of *th1* gene knock down at 27 hpf. RNA expression levels of zebrafish *th1* transcripts are presented as a percentage relative to the expression of the zebrafish housekeeping gene *elf1a*. [F2,8 = 4.9 p<0.05] Values are mean±SEM. * p<0.05.

**Figure 3:**
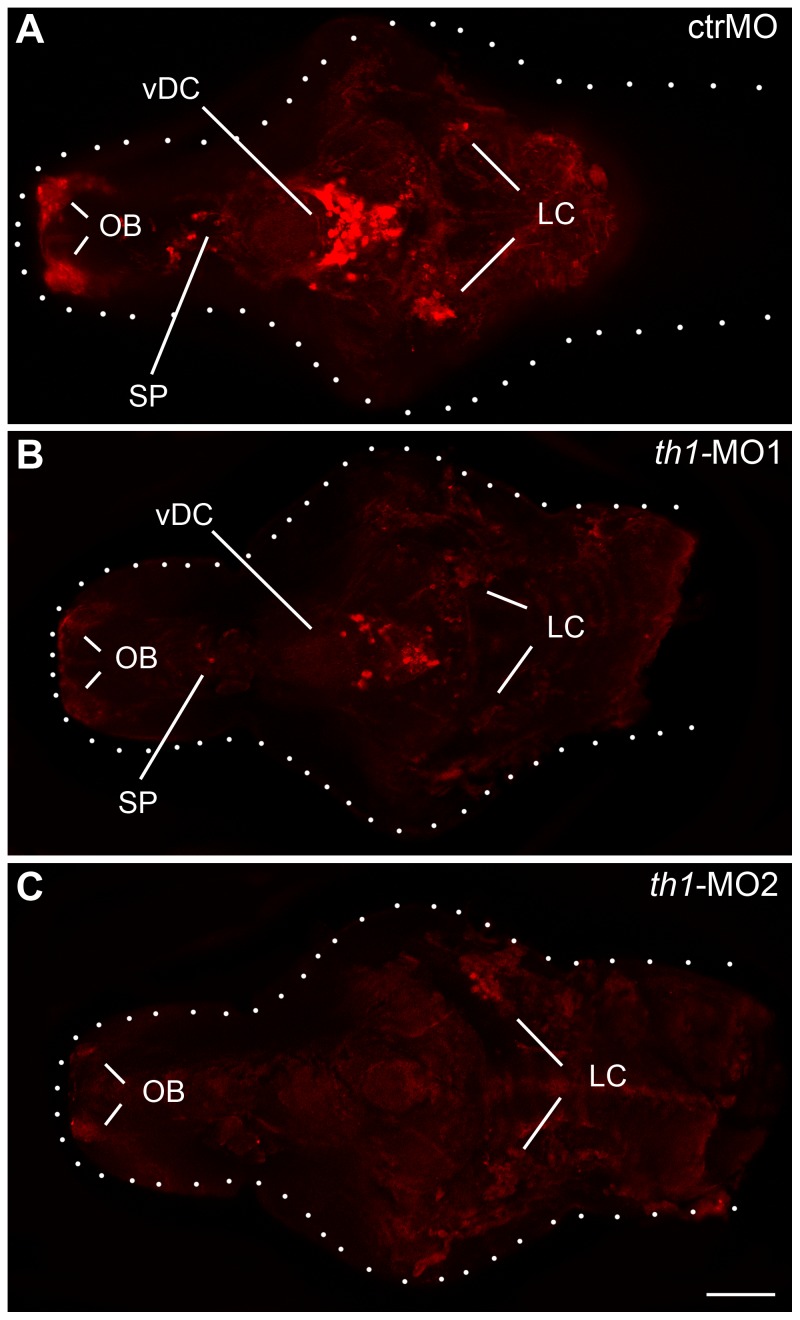
Altered patterning of TH-positive cells in the larval brain of *th1* morphants. Immunofluorescence of TH-containing cells in the brains of control MO (A), *th1*-MO1 (B) and *th1*-MO2 (C) injected embryos (6 dpf). Confocal z-projections of larval brains are shown from a dorsal perspective, anterior to the left. OB olfactory bulb, LC locus coeruleus, SP subpallium, vDC ventral diencephalon. Dotted lines indicate brain outline. Scale bar = 100 µm.

**Figure 4:**
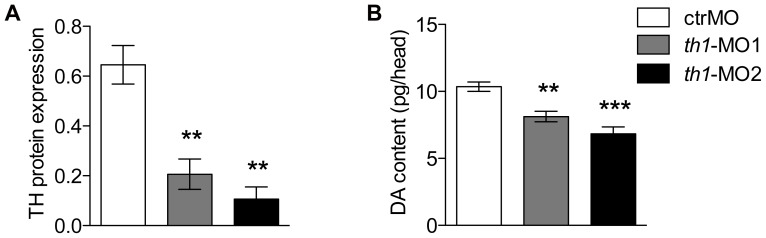
TH protein and dopamine content are reduced in *th1* morphant larvae. (A) Western blot reveals the expression of TH1 protein is significantly reduced in *th1*-MO1 and *th1*-MO2 injected embryos compared with control MO (6 dpf) [F2,8 = 20.4 p<0.01]. Protein expression levels are presented as a percentage relative to the expression of α-tublin (B) Dopamine (DA) content is significantly reduced in *th1*-MO1 and *th1*-MO2 injected embryos compared with control MO (6 dpf) [F2,11 = 17.5 p<0.01]. Values are mean±SEM. **p<0.01, ***p<0.001.

**Figure 5:**
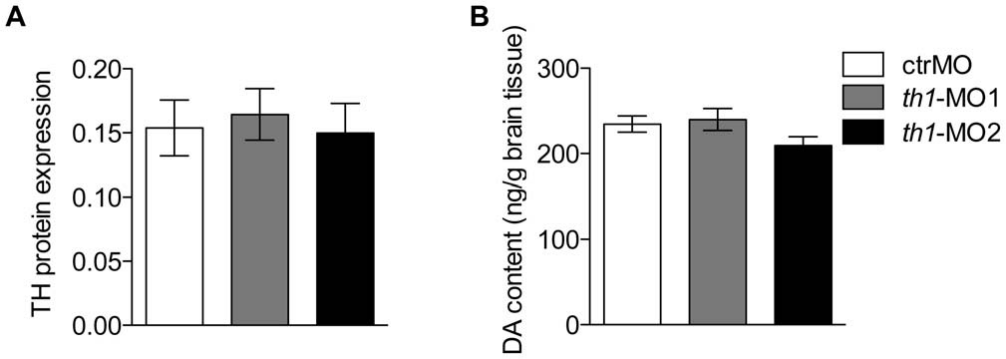
TH protein expression and dopamine content return to normal by adulthood. Western blot and HPLC show that zebrafish TH1 protein (A) and synthesis of DA (B) return to control levels in both th1-MO1 and th1-MO2 adult morphants. TH protein expression levels are presented as a percentage relative to the expression of α-tublin.

Treatment with *th1*-MO1 and *th1*-MO2 reduced *th1* mRNA (ENSDARG00000030621) transcript expression in zebrafish embryos at 27 hpf (pooled n = 30). However this reduction was only significant in *th1*-MO2 morphants [F2,8 = 4.9 p<0.05] (Figure 2). To investigate the effect of *th1* knock down on the distribution of TH positive cells in the zebrafish larval brain we used immunofluorescence and confocal microscopy at 6 dpf. In the *th1*-MO1 morphant brain we observed reduced TH immunoreactivity (TH-ir) in the olfactory bulb, subpallium, the ventral diencephalon, including the posterior tuberculum, the lateral and dorsal hypothalamus, and the locus coeruleus. *th1*-MO2 morphants displayed a complete loss of TH-ir cell groups in the subpallium and ventral diencephalon. A small number of TH-ir cells were present in the olfactory bulb and the locus coeruleus (Figure 3). To confirm this morphant-induced pattern of reduced TH expression, we investigated corresponding TH protein levels by western blot. TH protein levels were significantly reduced in both *th1*-MO1 and *th1*-MO2 morphants to only 33% and 18% of those seen in control larvae [F2,8 = 20.4 p<0.01] (Figure 4A). Measurements of the DA content also revealed significant but more modest MO-induced reductions to 78% and 66% of those observed in control larvae [F2,11 = 17.5 p<0.01] (Figure 4B). As expected, due to the transient character of MO-induced gene knock-down, both TH protein and DA neurotransmitter levels had returned to normal by the time fish were behaviourally tested at adulthood (Figure 5).

### Loss of *th1* gene function results in impaired behaviour in the adult fish

**Figure 6:**
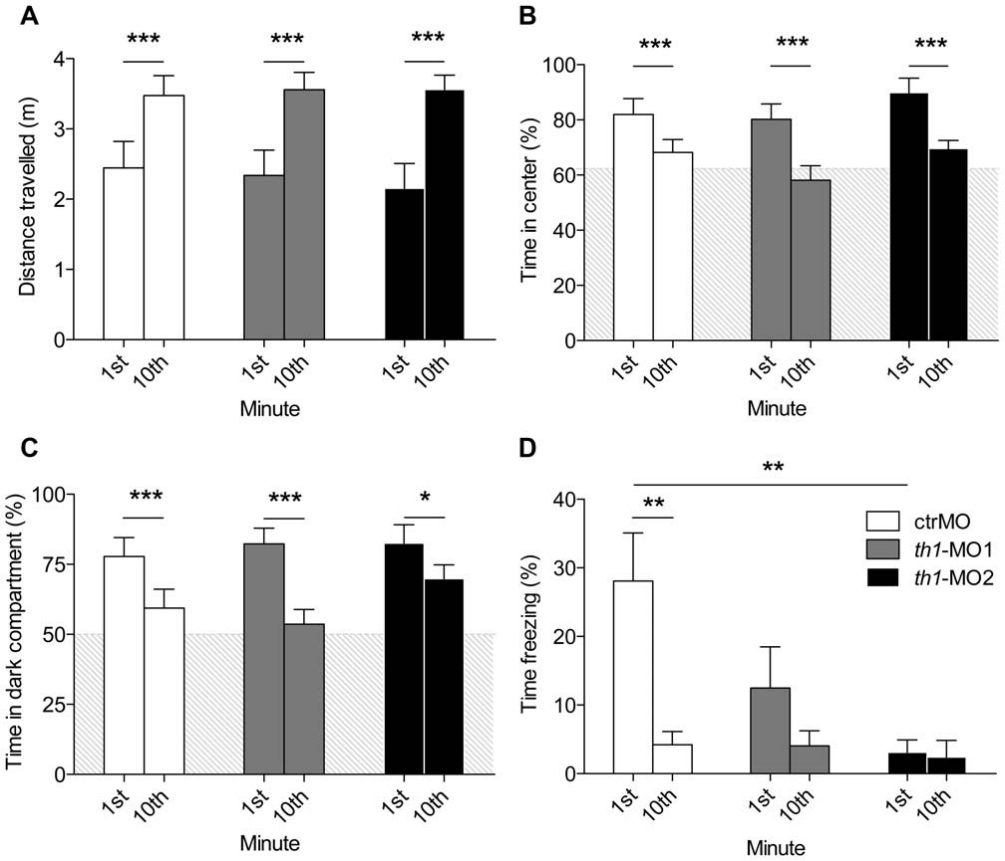
Adult morphants show normal locomotor and habituation but impaired freezing behaviour. Locomotor activity was measured as (A) distance travelled in metres in the open field tank, (B) time spent in the centre of the open field tank, (C) time spent in the dark compartment of the place preference tank, and (D) Percent time fish spent freezing in the open field tank. Habituation within these paradigms was assessed by comparing swimming behaviour in the 1^st^ minute with the 10^th^ minute. Shaded areas represent percentage area of tank defined as centre (open field tank) or dark (place preference tank). Freezing behaviour was abolished in both *th1*-morphants. (repeated measures ANOVA followed by Bonferroni's test) *p<0.05, **p<0.01, ***p<0.001.

Distance travelled, thigmotaxis and scototaxis did not differ among groups of control, *th1*-MO1 and *th1*-MO2 fish. All fish travelled significantly less during the 1^st^ minute (2.3±0.15 m) compared with the 10^th^ minute of testing in the open field tank (3.52±0.05 m) [F1,67 = 30.4 p<0.001] (Figure 6A). They also spent significantly more time in the centre of the open field tank (Figure 6B) and in the dark compartment of the place preference tank (Figure 6C) during the 1^st^ minute than the last minute (centre: [F1,67 = 26.3 p<0.001]; dark compartment: [F1,50 = 15.8 p<0.001] respectively). Control and MO-exposed fish steadily increased their horizontal exploratory activity over the 10-min trial (Figure 6A–6C).

Zebrafish when placed into a novel tank-environment display normal habituation by first freezing followed by a gradual increase in exploration [23]. Accordingly control fish spent a great proportion of time freezing during the 1^st^ minute (28%) compared with the 10^th^ minute (4%) [F1,68 = 7.9, p = 0.006] in the open field tank. This behaviour was abolished in the morphant fish. *th1*-MO2 fish froze significantly less [F2,68 = 3.8, p = 0.03] during the 1st minute than the controls. By the end of the trial all fish presented comparable amount of freezing (3%±1%) (Figure 6D).

**Figure 7:**
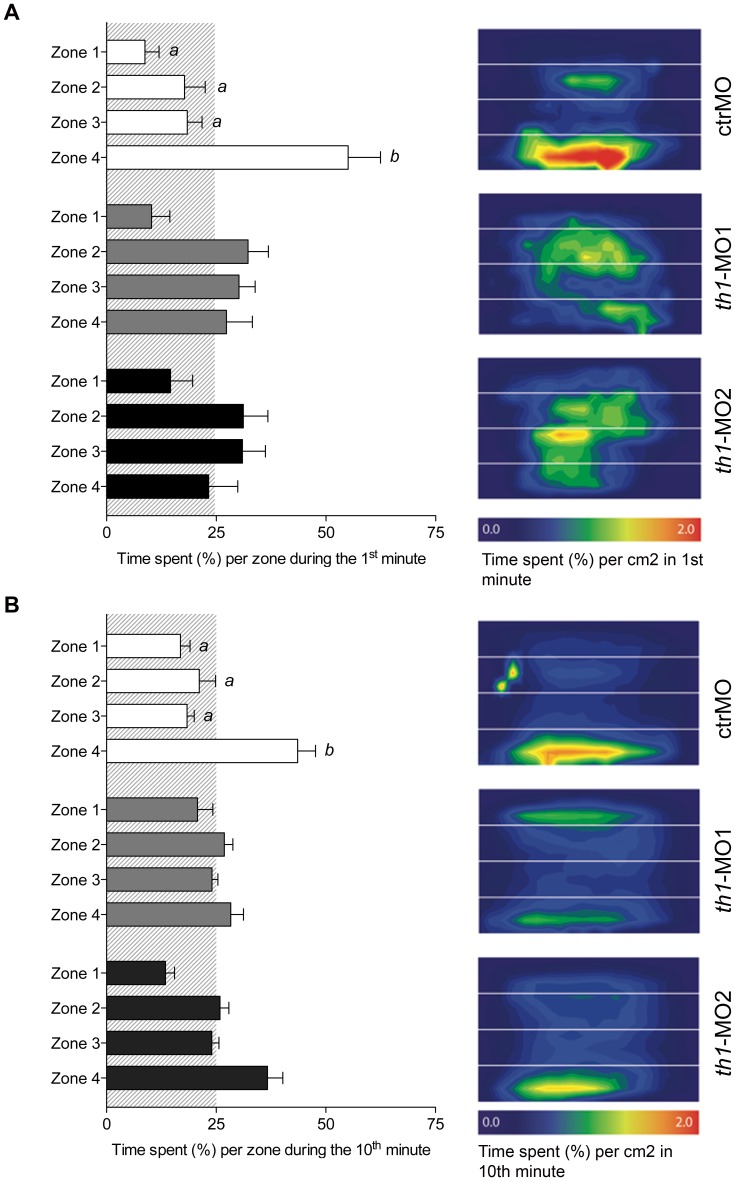
Adult morphants have impaired diving behaviour in a novel environment. Early disruptions of *th1* expression have long-lasting effects on the vertical diving behaviour in the novel diving tank task. Diving activity was assessed as the percentage time that was spent in each vertical zone (1–4) during a 10-minute trial. (A) shows behaviour that was displayed during the 1^st^ minute, and (B) shows behaviour during the 10^th^ minute. Letters indicate statistically significant differences between groups (one-way ANOVA followed by Bonferroni's test), p≤0.05. Corresponding heat maps are displayed to reflect fish location in each zone.

The novel diving test evaluates a fish's vertical exploratory activity based on its instinct to dive to the bottom in a novel environment and over time gradually explore upper areas of the tank. While the control fish displayed this behaviour, it was entirely abolished in both *th1*-MO1 and *th1*-MO2 morphants. Controls spent significantly more time in the bottom of the tank (zone 4) during both the 1^st^ [F3,69 = 12.9 p<0.001] and final 10^th^ minute of the trail [F3,69 = 12.5, p<0.001]. However, no zone preference was displayed by either of the MO-treated fish during either the 1^st^ or the 10^th^ minute of testing (Figure 7).

## Discussion

Our data provide evidence that transient reductions in TH during zebrafish development produce persistent effects on the behaviour of those fish as adults. Certain behaviours typically associated with abnormalities in DA signaling, such as locomotion and place preference, were normal in these fish indicating that our intervention did not produce any gross or permanent dopaminergic lesion. The fact that TH protein levels and DA content had returned to normal by the time these fish were behaviourally examined supports this interpretation. However, MO-treated fish showed abnormal behavioural responses to novel environments, with reductions both in freezing and bottom-dwelling. Together these measures indicate a potential anxiolytic phenotype in *th-1* morphants. It is interesting therefore to note that scototaxis, behavior, another potential indicator of anxiety in fish was normal. These behaviours however have recently been studied in detail and shown to be dissociable although the neurochemical mechanisms behind each individual behavior are poorly understood [41].These findings suggest that transient, early reductions in DA synthesis change brain development in a way that cannot be completely compensated for in the adult. The developmental mechanisms responsible for these abnormal adult behaviours remain unknown.

The embryonic and larval zebrafish catecholamine system has been described previously in detail [42], [43], [44], [45]. As early as 16 hpf, catecholamine precursor cells become post-mitotic and differentiate into DA neurons in the ventral diencephalon or noradrenergic neurons in the locus coeruleus. Between 24 and 48 hpf, the first longitudinal and commissural TH-positive axons can be detected. By 3 dpf, most of the DA cell groups and circuits described in the adult brain are well established [44], [45], with DA cell clusters formed in the olfactory bulb, pretectum, retina, and the ventral diencephalon including the posterior tuberculum, thalamus and hypothalamus. By 5 dpf DA post-synaptic receptor targets in these structures are all well established [46], [47]. Here we show that MO-mediated knock down of *th1* expression significantly reduces levels of *th1* mRNA, TH protein and DA in a consistent MO-related pattern until at least 6 dpf. In the adult, the neurotransmitter DA is responsible for mediating a host of behaviours from locomotion to attention and memory. In the developing brain it also acts as an extrasynaptic signaling molecule for axon guidance and neuron migration [48]. Therefore, we consider it likely that the reduction in DA synthesis seen in MO-exposed larval zebrafish may have affected early events in DA neuron connectivity. Such early alterations may underlie the abnormal behaviours reported here for MO-exposed adult fish.

While both morphants showed a strong decrease in TH protein levels at 6 dpf, corresponding measures of DA content in larval heads were not reduced to the same extent. One explanation for this is compensation from a paralog of the *th1* gene. Gene duplication, the generation of two or more paralogs of one gene, has been shown in the zebrafish lineage for a number of genes [49], [50], [51]. Duplication for the *tyrosine hydroxylase* gene, *th2*, in zebrafish was first described in 2005 [52]. The distribution of *th2* has been subsequently mapped using *in-situ hybridization* [53] and, although *th2* distribution is widely distributed in the adult zebrafish brain, the level of its expression in the larval brain is much lower than that of *th1*. Therefore, the co-expression of *th2* in larval brain may be capable of partial but not full compensation for the experimentally induced reduction of *th1* as it relates to DA synthesis. Nevertheless, DA synthesis was significantly reduced in a MO-dependent fashion in larval brain and this early reduction in DA synthesis was sufficient to produce long-term alterations in adult behaviour.

The assessment of adult zebrafish behaviour is now an established practice [54], [55], [56], [57], The natural tendencies to freeze or seek protection at the bottom of the testing apparatus when exposed to a novel environment have been well documented in adult zebrafish [18], [19], [21], [25], [26], [58]. These behaviours are controlled by a combination of microcircuits and a wide variety of neurotransmitters. For example, gamma-amino butyric acid (GABA) antagonists and agents that increase serotonin levels have been shown to produce the exact phenotype reported here with decreased freezing and abolished preference for the bottom zone of a diving tank apparatus. In contrast, sedatives such as pentobarbital and agents that increase DA such as cocaine increase freezing and enhance time spent at the bottom zone of a novel diving tank [15], [16], [40]. Indirect confirmation for the role of DA in these behaviours comes from the observations that after withdrawal of the DA agonist cocaine, increased bottom dwelling behaviour of zebrafish could be observed [16]. There may even be some interplay between discrete neurotransmitter systems governing these behaviours because Souza (2011) and colleagues have demonstrated that the use of exogenous DA agonists and antagonists during the very early stages of development disrupts GABAergic development and larval motor behaviour [59]. These authors also showed that reduced DA signaling in 3–5 dpf larvae preceded the loss of GABAergic neurons [59]. It will now be interesting to characterize these neurotransmitter systems in *th1* morphants as both larvae and adults.

The zebrafish is an increasingly important model in which to study the neurological bases of psychiatric disorders [60]. Recently, MO knock-down of *lphn3.1*, an endogenous candidate susceptibility gene for attention deficit hyperactivity disorder (ADHD) was described. Although the exact function of this gene is unknown, these authors show that the transient silencing of this gene during larval development selectively affects dopaminergic systems and larval behaviour [61]. It is also interesting to compare our results with those of others studying adult behaviour in zebrafish with compromised dopaminergic development. NR4A2/Nurr1 is an essential specification factor for DA neurons in both mammals [62] and zebrafish [30]. When the expression of NR4A2/Nurr1 is knocked down in zebrafish larvae, the production of both TH and DA are reduced to a similar level to that shown here. Although a broad behavioural battery was not employed in that study, depending on the MO used, early knock-down of NR4A2/Nurr1 produced permanent effects on adult fish locomotion [30]. The differences between these behavioural effects and the ones that we observe may result from broader effects of this specification factor on other dopaminergic elements, such as its effects on DA release mediated by the DA transporter [62] as compared to the more selective reduction in DA synthesis studied here.

## Conclusions

Developmental psychiatric conditions such as schizophrenia are widely believed to involve abnormalities in DA signaling. Based on work in rodents, we have proposed that early alterations in DA ontogeny may precede the onset of these serious psychiatric conditions [13]. Here we have examined whether transient changes in DA synthesis during early stages of brain development in zebrafish lead to altered brain function in adults. We show that transiently decreasing DA synthesis during the early stages of development changes the adults' responses to novel situations. The exact neurochemical and anatomical correlates of this behaviour now need to be characterized. Given the experimental advantages of the zebrafish, these results lay the groundwork for further studies into the anatomical, circuit, and physiological underpinnings of these behavioural changes [60].
